# Successful In Vitro Expansion and Differentiation of Cord Blood Derived CD34+ Cells into Early Endothelial Progenitor Cells Reveals Highly Differential Gene Expression

**DOI:** 10.1371/journal.pone.0023210

**Published:** 2011-08-12

**Authors:** Ingo Ahrens, Helena Domeij, Denijal Topcic, Izhak Haviv, Ruusu-Maaria Merivirta, Alexander Agrotis, Ephraem Leitner, Jeremy B. Jowett, Christoph Bode, Martha Lappas, Karlheinz Peter

**Affiliations:** 1 Department of Cardiology and Angiology, University Hospital Medical Centre, Freiburg, Germany; 2 Atherothrombosis and Vascular Biology, Baker IDI Heart and Diabetes Institute, Melbourne, Australia; 3 The Blood and DNA Profiling Facility, Baker IDI Heart and Diabetes Institute, Melbourne, Australia; 4 Department of Immunology, Alfred Medical Research and Education Precinct, Monash University, Melbourne, Australia; 5 Department of Obstetrics and Gynaecology, Mercy Hospital for Women, University of Melbourne, Melbourne, Australia; University of São Paulo, Brazil

## Abstract

Endothelial progenitor cells (EPCs) can be purified from peripheral blood, bone marrow or cord blood and are typically defined by a limited number of cell surface markers and a few functional tests. A detailed in vitro characterization is often restricted by the low cell numbers of circulating EPCs. Therefore in vitro culturing and expansion methods are applied, which allow at least distinguishing two different types of EPCs, early and late EPCs. Herein, we describe an in vitro culture technique with the aim to generate high numbers of phenotypically, functionally and genetically defined early EPCs from human cord blood. Characterization of EPCs was done by flow cytometry, immunofluorescence microscopy, colony forming unit (CFU) assay and endothelial tube formation assay. There was an average 48-fold increase in EPC numbers. EPCs expressed VEGFR-2, CD144, CD18, and CD61, and were positive for acetylated LDL uptake and ulex lectin binding. The cells stimulated endothelial tube formation only in co-cultures with mature endothelial cells and formed CFUs. Microarray analysis revealed highly up-regulated genes, including LL-37 (CAMP), PDK4, and alpha-2-macroglobulin. In addition, genes known to be associated with cardioprotective (GDF15) or pro-angiogenic (galectin-3) properties were also significantly up-regulated after a 72 h differentiation period on fibronectin. We present a novel method that allows to generate high numbers of phenotypically, functionally and genetically characterized early EPCs. Furthermore, we identified several genes newly linked to EPC differentiation, among them LL-37 (CAMP) was the most up-regulated gene.

## Introduction

Endothelial progenitor cells (EPCs) represent a group of circulating cells derived from CD34+ hematopoietic stem cells (HPC). They are thought to stimulate angiogenesis either by their ability to differentiate into mature endothelial cells or by stimulating the formation and repair of the endothelium and vessel formation via paracrine stimuli [1], [2], [3], [4], [5], [6].

Lately, the use of EPCs as a potential therapeutical tool for treatment of cardiovascular disease (CVD) has drawn much interest [5], [7], [8]. A number of *in vivo* studies hypothesized that EPCs posses the ability to repair damaged myocardial tissue, as the injection of EPCs into both human and animal failing hearts have shown to improve left ventricular function [5], [8], [9], [10]. However, the mechanisms responsible for this phenomenon are yet to be unravelled. The most widely used phenotypic characterization for EPCs includes expression of CD34 and VEGFR-2 (KDR, CD309) [2], [3], [4], [11], [12] in addition to their ability to take up acetylated-LDL and to bind ulex lectin [1], [3], [13]. The functional capacity of EPCs is most often described by their ability to form colony like structures when cultured on fibronectin and their ability to support the formation of tubule-like structures in Matrigel™ [1], [14].

The general term EPC was built on the initial description of a rare population of cells with the capability to contribute to the formation of new blood vessels and regeneration of damaged endothelium [1]. A recently evolving and ongoing discussion of the different culture and isolation techniques, which have been used to generate EPCs, led to the conclusion that the general term EPCs describes a heterogenous population of cells that, according to isolation, culture and characterization techniques, display different phenotypes and functions [2], [3], [13], [15]. More appropriate and increasingly accepted definitions aim to dissect the general term EPC into at least two different populations of cells: early EPCs (also described as pro-angiogenic cells) [2] and late EPCs, also described as endothelial outgrowth cells (OEC) or endothelial colony forming cells (ECFC) [3], [13], [16]. The culture techniques applied throughout our *in vitro* study and the phenotype and functional capacities of the putative EPCs generated from *in vitro* expanded CD34+ cord blood mononuclear cells resemble most likely early EPCs. Therefore, we use the term early EPC to describe the cells generated in our study and the more general term EPC when referring to other studies that did not explicitly distinguish early and late EPCs.

The common barrier for the characterization and subsequent utilization of putative EPCs is the poor number of cells obtained after purification from peripheral or cord blood. EPCs represent a very small subset of peripheral blood mononuclear cells, ranging from 0.002 to 0.01% in peripheral blood and 0.2–1% in umbilical cord blood [12]. According to the cell numbers that have been used for systemic infusion of allogenic EPCs in patients [17], [18], this would have required a significant amount of blood if the cells would not have been expanded in vitro before [5].

Herein we describe a novel method that allows for the generation of a high cell yield of well-defined and functionally active early EPCs derived from CD34+ cord blood cells, which could be used for *in vitro* and *in vivo* studies. Furthermore, by the use of microarray-based gene expression profiling and quantitative PCR we have identified a number of genes, that may play a central role in the differentiation process of hematopoietic progenitors to early EPCs.

## Materials and Methods

### Isolation of CD34+ cells

Mononuclear cells (MNCs) were isolated from human umbilical cord blood (HUCB) obtained from healthy donors following normal full term deliveries after their written-informed consent. Ethics approval was granted by the Human Research Ethics Committee, Mercy Health, Mercy Hospital for Women, Melbourne, Australia (Project number R08/24).

HUCB was collected in 50 ml Falcon tubes (BD Bioscience, NJ, USA) containing 15 ml of the anticoagulant citrate phosphate dextrose. After collection, HUCB was diluted 1∶3 in isolation buffer (PBS, 0.1% BSA, 0.6% citrate, pH 7.4) and MNCs were isolated from the diluted HUCB by density gradient centrifugation, where 20 ml of diluted HUCB was layered onto 15 ml Ficoll (GE Healthcare, Uppsala, Sweden) and centrifuged for 30 min at 800×*g*. Thereafter the interphase containing MNCs was collected, followed by two washing steps, 20 min at 500×*g* and five min at 400×*g*, respectively. The washed MNCs were then subjected to magnetic beads-based selection of CD34+ cells using Dynal® CD34 Progenitor Cells Selection System (Invitrogen, Oslo, Norway) following the manufacturer's recommendation. The number of positively selected CD34+ cells, the hematopoietic progenitor cells (HPC) was assessed in a Neubauer haemocytometer and the purity of the CD34+ cells was evaluated by flow cytometry.

### Expansion of CD34+ HPCs

The cord blood derived CD34+ HPCs were cultured at a density of 3–5×10^4^ cells/400 µl/1.8 cm^2^ in a humidified incubator at 37°C with 5% CO_2_. The cells were cultured for seven days in serum free StemSpan® medium (StemCell Technologies, Vancouver, Canada) during the initial expansion period and supplemented with 1% penicillin-streptomycin (Sigma-Aldrich, St. Louis, USA) and recombinant human (rh) Flt-3 ligand (100 ng/ml), rh stem cell factor (100 ng/ml), rh IL-3 (20 ng/ml), rh IL-6 (20 ng/ml), all purchased from StemCell Technologies. The cell number after expansion was assessed in a Neubauer haemocytometer. The viability of the cells was determined by flow cytometry using FITC-labelled Annexin V (Invitrogen, Carlsbad, USA) following the manufacturers recommendations (Figure S2).

### Differentiation of HPCs to endothelial progenitor cells (EPCs)

After the seven day expansion period, the HPCs were collected and counted. Thereafter the conditioned medium was removed and the cells were cultured (3×10^5^–1×10^6^/1.5 ml/9.6 cm^2^) in endothelial cell growth medium-2 (EGM-2) containing FBS (2%), hydrocortisone, hFGF, VEGF, R^3^-IGF-1, ascorbic acid, hEGF, gentamycin, amphotericin-B and heparin (Lonza, Basel, Switzerland). After three days of culture, the cells were collected and transferred to plates coated with fibronectin (10 µg/ml) (Sigma-Aldrich, St. Louis, USA) at a density of (1×10^6^ cells/1.5 ml/9.6 cm^2^) and cultured for an additional three days in fresh EGM-2 medium.

### Ulex-lectin binding and uptake of acetylated LDL

After three days of culture in medium, HPCs were seeded onto a fibronectin coated 24 well plates at a density of 3×10^5^ cells/0.5 ml/1.8 cm^2^ and cultured in EGM-2 for three days, as described above. The cells were then washed twice with PBS at 37°C, and incubated with Dil-AcLDL (6 µg/ml) (Invitrogen, Carlsbad, USA) for 1 h at 37°C in the dark. Thereafter, the cells were washed twice with PBS at 37°C and incubated with FITC-conjugated ulex lectin (10 µg/ml) (Sigma-Aldrich, St. Louis, USA) for one h at 37°C in the dark. After two final washing steps with PBS at 37°C, the cells were fixed with 0.3 ml CellFIX solution (BD Biosciences, NJ, USA), and subsequently analysed for uptake of Dil-AcLDL and binding of FITC-ulex lectin using an Olympus 1X81 inverted fluorescence microscope.

### Flow cytometry

Cells were analyzed for the expression of a selection of cell surface markers after both expansion and after differentiation. Human umbilical vein endothelial cells (HUVECs) were analysed at passage four. The cell surface markers included CD34, CD309 (VEGFR-2), CD144 (VE-Cadherin), CD18, CD61 and CD45. In addition cells were analysed for the expression of CD34 directly after the purification procedure. In brief, the cells were incubated with FITC-conjugated CD34 (clone QBEND/10, Chemicon), CD18 (clone C71/16, Beckman Coulter), CD45 (clone HI30, BD Pharmingen), CD61 (clone SZ21, Beckman Coulter) and PE-conjugated CD144 (clone 123413, R&D systems) or CD309 (clone 89106, R&D systems) in PBS for 15 min in dark at room temperature. The cells were then washed in PBS and fixed using 1× Cellfix (BD Biosciences). A negative control with an isotype-matched antibody was included in each run. The cell surface expression was analysed in a FACSCalibur™ flow cytometer using CellQuest software (Becton and Dickinson, San Jose, CA, USA). Between 10,000–20,000 events per test were acquired.

### Colony forming unit assay

Culture of CFU-Hill colonies (StemCell Technologies) were performed according to the manufacturer's recommendations with the exception that we used ten times less cells (5×10^5^ cells/well) and *in vitro* differentiated cord blood derived early EPCs instead of freshly prepared MNCs. In brief, after the differentiation period of 72 h on fibronectin in EGM-2 media, the cells were collected and counted. The collected cells were cultured (5×10^5^ cells/1.5 ml/9.6 cm^2^) on fibronectin pre-coated six well plates (BD Biosciences) in EndoCult® Liquid Media containing EndoCult Supplements (StemCell Technologies). After two days, the non-adherent cells were collected and transferred to fibronectin pre-coated 24-well plates (BD Biosciences) at a density of 3×10^5^ cells/1 ml/1.8 cm^2^. After a further three days of culture, the cells were washed twice with PBS, fixed with methanol, and the colonies were visualised with Giemsa staining (Invitrogen Gibco, Carslbad, USA), following the manufacturer's recommendations. The number of colonies per well was counted with an inverted microscope (Olympus CKX41). In some of the colony assays the uptake of Dil-AcLDL and the binding of FITC-labelled Ulex-lectin was determined as described previously using fluorescence microscopy (Olympus 1X81).

### Endothelial tube formation assay

The capability of the putative EPCs to support endothelial tube formation was assessed in a co-culture system with human umbilical vein endothelial cells (HUVECs) (passages P3–P5) using Matrigel™ (BD Biosciences,). In brief, wells of a 96-well plate were coated with 50 µl ice cold Matrigel™ followed by incubation at 37°C for one hour. Thereafter, 100 µl EGM-2 medium containing 25,000 HUVECs and 100 µl EGM-2 containing 25,000 putative EPCs were added to the Matrigel™. Incubation was carried out for 16 hours in a humidified atmosphere at 37°C with 5% CO_2_. Tube formation was assessed with an inverted microscope (Olympus 1X81) and Cell∧P Imaging Software (Olympus). Digital photomicrographs of each single well were taken at a four times magnification and the total number of tubes, the branching points, the length of the tubes as well as the sum of the lengths of the tubes were calculated for each well. In some experiments, the putative EPCs were traced in the Matrigel™ by pre-staining the EPCs with cell-tracker green as described below (adhesion assay).

### Microarray Analysis

Total RNA was extracted from 2×10^6^ cells before and after 72 h culture on fibronectin (10 µg/ml). The RNA was obtained using Qiagen® RNeasy protect mini kit™ following the manufacturer's instructions. RNA concentration and integrity was analysed by NanoDrop (Thermo Fisher Scientific, Waltham, USA) and MultiNA microchip electrophoresis (Shimadzu Biotech, Japan) according to the manufacturer's recommendations. Total RNA was amplified with the TotalPrep™ RNA Amplification Kit (Ambion, UK) and applied to Illumina® Human WG-6 v3.0 Expression BeadChip kits according to the manufacturer's instructions. Fluorescent bead intensity was transformed into gene expression level via Illumina Genome Studio, including quantile normalisation [19] and background subtraction. The exported reports were analysed on GeneSpring GX10, Partek GS, and arraytools (http://linus.nci.nih.gov/pub/rsimon/ArrayTools). Genes with less than raw signal <250 and a detection confidence >0.8 in at least three arrays were filtered out, leaving 6,064 genes for further analysis. Whisker Box plot was used to confirm the quantile normalisation eliminated systematic cross array variations in overall dynamic range. Quality control of the samples and controlling for systematic bias was performed using principal component analysis, and unsupervised hierarchical clustering to show that samples segregated according to treatment groups. Differentially expressed genes were selected based on a bayesian “volcano plot” of expression fold change greater than two fold, and significance P value <0.05, including Benjamini Hochberg false discovery correction. Differential expression of the chosen genes across the treatment groups was assessed using supervised hierarchical clustering [20], that measures proximity of distribution of samples and genes. The interpretation of the resulting gene lists was performed using gene ontology web interface (http://david.abcc.ncifcrf.gov/), Gene Set Analysis [21], protein-protein interaction KEGG database [22], and by Ingenuity Pathways Analysis [23].

### Quantitative Real Time PCR analysis

First-strand cDNA was synthesized using 50 ng of total RNA, obtained as described in the microarray section, and random primers in a 10 µl reverse transcriptase reaction mixture using Invitrogen's Superscript cDNA synthesis kit (Invitrogen) following the manufacturer's recommendations.

Quantitative real-time PCR assays were carried out with Applied Biosystems 7500 Real-Time PCR system using SYBR® Green detection mix (Applied Biosystems, Carlsbad, USA). Primers were designed in-house and synthesized by GeneWorks (Hindmarsh, Australia). PCR amplification was performed in a 96-well plate with final volume of 20 µl reaction mixture in each well. Each sample was run in triplicates with 5 ng cDNA, 1× SYBER® Green mix, and 5 µM of the following primers for: CAMP sense 5′-CCAGGCCCACGATGGAT-3′ and anti-sense 5′-GCACACTGTCTCCTTCACTGTGA-3′; PDK4 sense 5′-CGCTGTCCATGAAGCAGCTA-3′ and anti-sense 5′-CCTTCAGAATGTTGGCGAGTCT-3′; A2M sense 5′-CAATGTTATGGGTACCTCTCTTACTGTT-3′ and anti-sense 5′-GTGATGTGCCTCTTCGTGTTCTT-3′; IL18BP sense 5′-TCCCAGCCCCCAGTGTT-3′ and anti-sense 5′-CAGGATAAGCTCAGCGTTCCAT-3′; CD9 sense 5′-TGCTGTTCGGATTTAACTTCATCT-3′ and anti-sense 5′-GAATCGGAGCCATAGTCCAATG-3′; SERPINF1 sense 5′-GAGTGCCTCCCGGATCGT-3′ and anti-sense 5′-ACTTTTCCAGAGGTGCCACAAA-3′; FLT3 sense 5′-TCTTTAAGCACAGCTCCCTGAAT-3′ and anti-sense 5′-TCCAGCTTGGGTTTCTGTCAT-3′; LGALS3 sense 5′-GGTGCCTCGCATGCTGATA-3′ and anti-sense 5′-TTGGAAATCTAAAGCAATTCTGTTTG-3′; IL-1β sense 5′-CTTTGAAGCTGATFFCCCTAAA-3′ and anti-sense 5′-AGTGGTGGTCGGAGATTCGTA-5′; GDF15 sense 5′-AAACATGCACGCGCAGATC-3′ and anti-sense 5′-CGGTCTTTTGAATGAGCACCAT-3′.

Relative expression of the genes was obtained using the differences in cycle threshold (C_t_) between the sample and 18S ribosomal RNA (ΔC_t_). The difference in gene expression for the compared samples was calculated (ΔΔC_t_) and the fold difference was calculated as 2^ΔΔCt^.

### Static adhesion assay

The ability of the putative EPCs to adhere to a monolayer of non-activated or TNFα activated HUVECs was studied in a 96 well plate static adhesion assay. In brief, the wells were coated with fibronectin (10 µg/ml) for one h at 37°C, HUVECs (10,000 cells/well) were then plated to the fibronectin coated wells and cultured in EGM-2 for 24 h. HUVECs were then washed with fresh EGM-2 medium and treated with TNFα (10 ng/ml) or PBS for one h. The HUVEC monolayer was blocked with PBS containing 1% BSA for one h at 37°C. In the meantime, EPCs, which had been cultured for 72 h on fibronectin were stained with 4 µM cell-tracker green (Invitrogen) in EBM-2 at 37°C for 30 min, followed by 30 min incubation at 37°C with EBM-2 only. The static adhesion assay was carried out by adding putative EPCs (10 000/well) to the HUVEC monolayer and incubating the cells in EGM-2 for one h at 37°C. Thereafter, the non-adherent cells were removed by washing three times with PBS and EPCs adhered to HUVECs were captured using an Olympus 1X81 inverted fluorescence microscope (20×) For each well, three random optical fields were taken and the number of adhered EPCs was calculated using Image-Pro Plus 6.0.

### Dynamic adhesion assay under conditions of continuous flow

Rectangular glass capillary tubes (VitroCom, Mountain Lakes, USA) were coated with fibronectin (10 µg/ml) for two h at 37°C. The HUVECs were placed in the capillary tube by capillary force and allowed to adhere for three h at 37°C, with a change of EGM-2 medium every h. The adherent HUVEC monolayer was treated with TNFα (10 µg/ml) or PBS for one h at 37°C. The HUVEC monolayer was then washed with PBS and blocked with 1% BSA in PBS for one h at 37°C and the flow experiment was carried out. One end of the capillary was connected via flexible tubing to a syringe pump (Harvard Apparatus PHD 2000, Holliston, USA) that controls the flow of fluid through the capillary tube and the other end of the capillary was connected to a reservoir that allows the application of fluids and cells to the flow chamber system. Putative EPCs stained with cell-tracker green (for details see static adhesion assay) at a concentration of 1×10^6^ cells/ml were perfused to the capillary with a shear rate of 50 s^−1^ for three min. Video recordings, three for each capillary, were then analyzed using Image-Pro Plus 6 and the number of EPCs adhering to the HUVEC monolayer was quantified by fluorescence microscopy (Olympus 1X81).

### Flow cytometry for intracellular detection of LL-37 (CAMP)

For flow cytometry analysis of LL-37 (CAMP), cells were washed twice with PBS and permeabilized with 250 µl of BD Cytofix/Cytoperm™ solution (BD Bioscience, NJ, USA) for 20 min at 4°C. This was followed by two washing steps with BD Perm/Wash™ buffer. Thereafter cells were incubated with the monoclonal anti-LL-37 (CAMP) antibody OSX12 (ab87701, (abcam, Cambridge, UK) for 30 min at 4°C. Cells were then washed twice with BD Perm/Wash™ buffer and incubated with a secondary Alexa Fluor 488 labeled goat-anti-mouse IgG (A11029, Invitrogen, Carlsbad, USA) for 30 min at 4°C. Finally, cells were washed again twice with BD Perm/Wash™ buffer and analyzed by flow cytometry as described above.

### Statistical analysis

All experiments were performed with EPCs from at least three different donors, respectively. Mean and SD was used for descriptive statistics. Statistical analysis were performed using Sigma Stat. Differences between means were assessed by analysis of variance (ANOVA) and post hoc test were carried out according to Bonferroni. P-values less than 0.05 were considered statistically significant.

## Results

### Expansion and differentiation procedure of human umbilical cord blood derived HPCs

**Figure 1:**
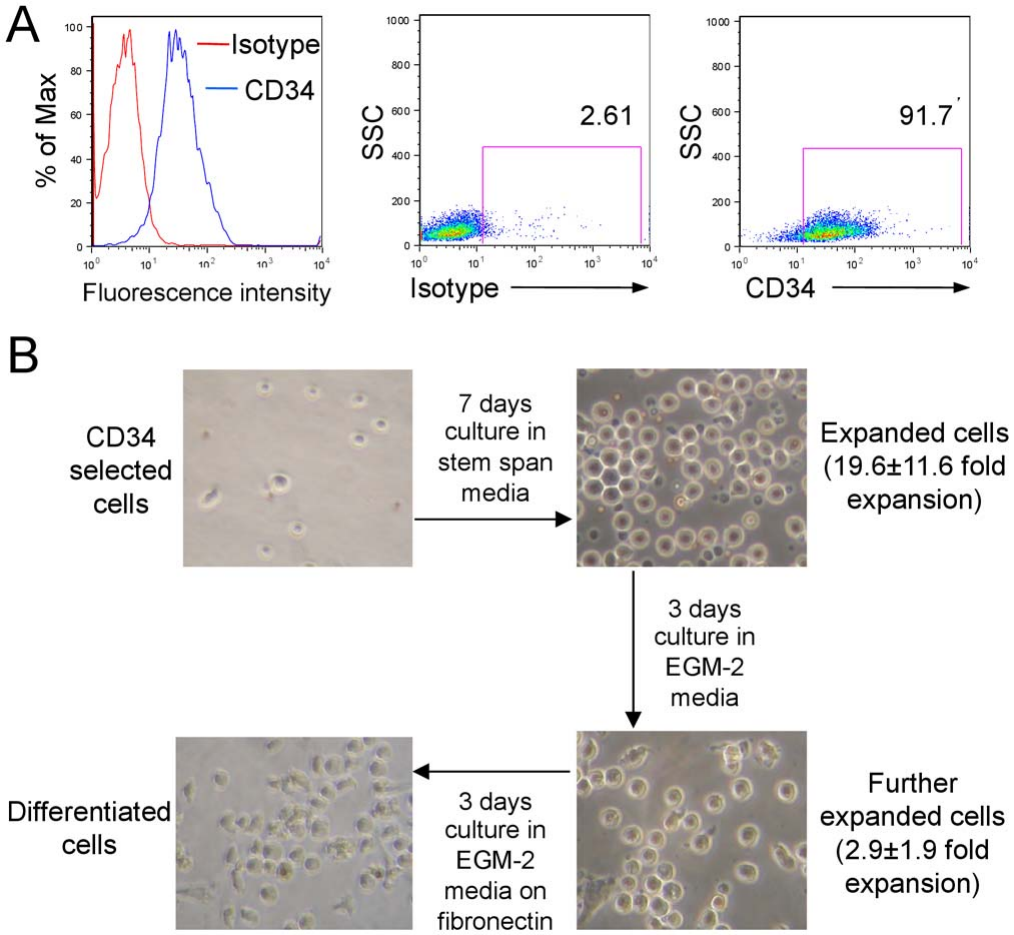
Isolation, expansion and differentiation of cord blood derived CD34+ HPCs. **A.** A representative histogram and dot plots showing CD34-expression after purification from cord blood depicted as fluorescence intensity values and percentage of CD34+ cells compared to the corresponding isotype control, respectively. **B.** The morphology and culture procedure for CD34+ cells after purification, seven days in expansion media, three days in medium and another three days in EGM-2 on fibronectin coated dishes. The fold expansion of cells represent mean and SD of n = 9.

The expansion and differentiation of cord blood derived CD34+ cells was undertaken in three subsequent steps over a time line of 13 days. After positive selection, single staining flow cytometry revealed that approximately 90% of the cells expressed CD34 after the positive selection process. A representative analysis is shown in Fig. 1A.

The selected cells were first cultured on non-treated culture plates in serum free StemSpan® medium supplemented with Flt-3 ligand, stem cell factor, IL-3 and IL-6. After seven days, the cells had expanded 20 fold at average (19.6±11.6, mean and SD, n = 9) and showed characteristics of a non-adhesive homogenously rounded group of cells (Fig. 1B). The expanded cells were then cultured for three days in endothelial cell growth medium-2 (EGM-2) resulting in an additional three-fold expansion (2.9±1.9, mean and SD, n = 9) still exhibiting a non-adhesive phenotype (Fig. 1B). After transferring these cells to fibronectin-coated wells and culturing for an additional three days in fresh EGM-2, there was a change in morphology to adherent more spindle like or oval shaped cells (Fig. 1B).

### Phenotypical characterisation of expanded HPC derived putative early EPCs

**Figure 2:**
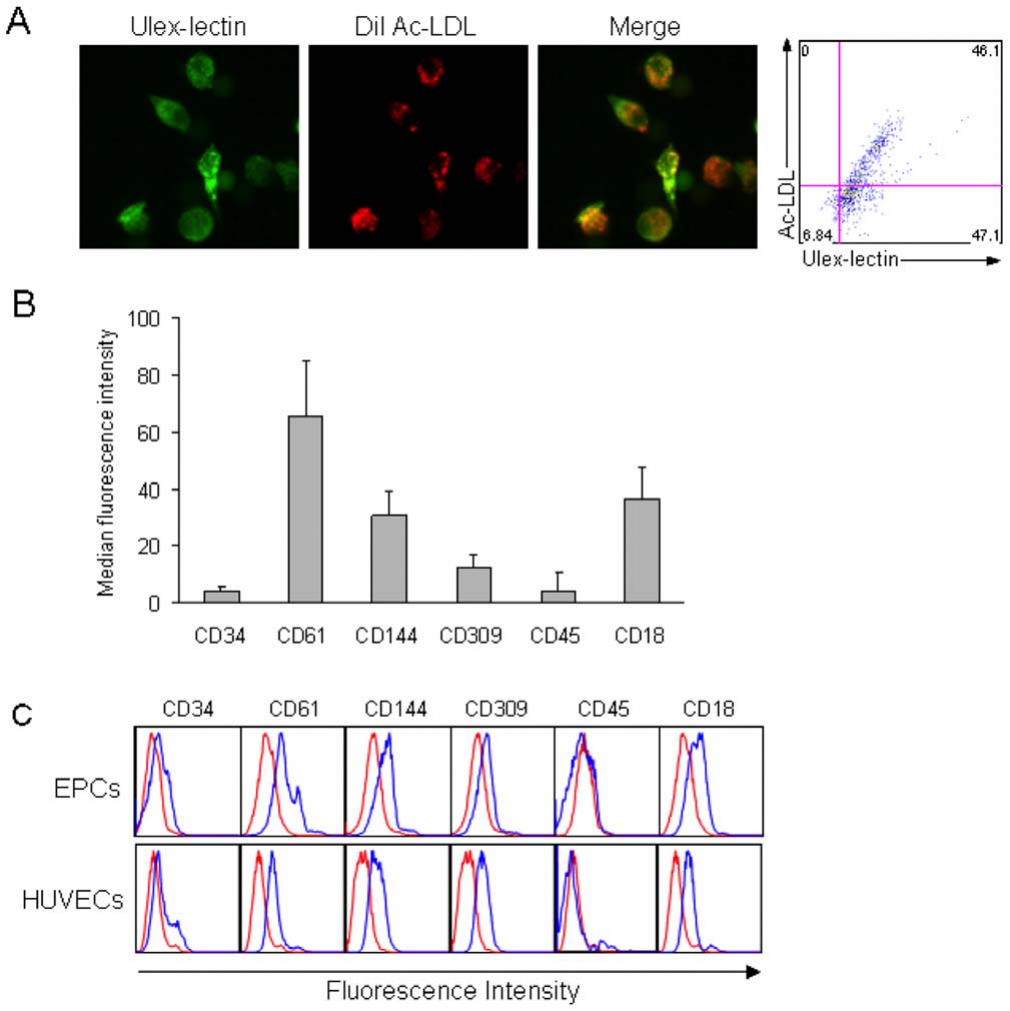
Phenotypical characterisation of expanded HPC derived early EPCs. **A.** Binding of ulex-lectin and uptake of acetylated LDL (AcLDL) on EPCs visualised with fluorescence microscopy and flow cytometry using FITC-labelled ulex-lectin and Dil-labelled acetylated LDL, respectively. **B.** Summary of median fluorescence intensity for CD34-FITC, CD61-FITC, CD144-PE, CD309-PE, CD45-FITC and CD18-FITC of five donors expressed as mean and SD compensated for respective isotype control. **C.** Representative histograms comparing fluorescence intensity on early EPCs and HUVECs of the cell surface markers for CD34, CD61, CD144, CD309, CD45 and CD18 (blue line) and corresponding isotype control (red line).

Expanded and differentiated HPCs were examined for EPC phenotype by the uptake of acetylated LDL (acLDL) and ulex lectin binding in addition to the detection of cell surface markers by flow cytometry. After 72 h culture on fibronectin, immunofluorescence microscopy and flow cytometry analysis showed a double positive staining for Dil-labelled AcLDL and FITC-labelled ulex lectin indicating that the expanded HPCs were differentiating towards EPCs. Representative photomicrographs are shown in Fig. 2A. Further flow cytometry analysis investigating the EPC markers VE-cadherin (CD144) and VEGFR-2 (CD309), revealed that early EPCs expressed CD144 and CD309, in addition to the β_3_-integrin subunit (CD61) and the β_2_-integrin subunit (CD18), whereas the expression of CD45 and CD34 was low (Fig. 2B). To further characterize the early EPC population, we compared the expression pattern of the early EPCs with human umbilical vein endothelial cells (HUVECs). As demonstrated in Fig. 2C, HUVECs showed similar expression patterns for CD144, CD309, CD61, CD18, CD45 and CD34.

### Functional characterization of putative early EPCs

**Figure 3:**
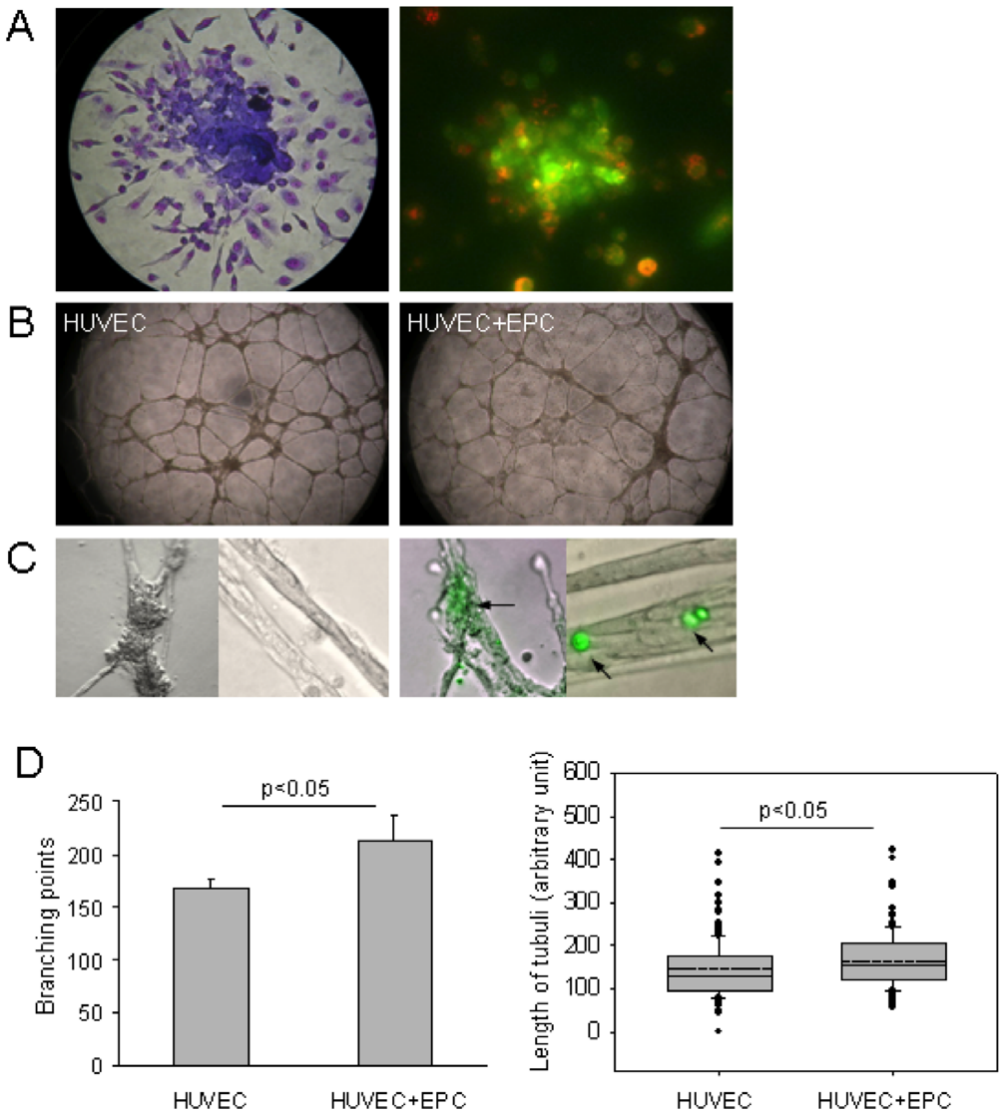
Functional characterization of putative early EPCs. **A.** A representative colony forming unit (CFU-Hill) visualised with giemsa staining and characterised with binding of FITC-labelled ulex-lectin and uptake of Dil-labelled acetylated LDL. **B.** Representative images of capillary network formed in a endothelial tube formation matrigel assay by HUVECs alone or HUVECs co-cultured with early EPCs. **C** Early EPCs pre-stained with cell tracker green (arrows) traced along the tubuli and in the branching area of capillary network **D.** The bar graph shows the total number of branching points (mean and SD, n = 3) and the box plot show the length of tubuli (dotted line represents the mean and the solid line represents the median, n = 488) in the endothelial tube formation matrigel with HUVECs alone or HUVECs co-cultured with early EPCs.

With the use of commercially available Endocult® Liquid medium kit from StemCell Technologies and fibronectin pre-coated plates, we demonstrated that early EPCs possess the ability to form CFU-Hill colonies (33±21 CFU/cm^2^, mean ± SD of n = 8, range 10–50). A representative colony is shown in Fig. 3A left panel. Cells in the centre of the colony stained predominately positive for ulex lectin binding and towards the periphery staining for uptake of acLDL increased (Fig. 3A, right panel).

The angiogenic property of the early EPCs was also studied in an endothelial tube formation assay, where early EPCs were either cultured alone or in co-culture with endothelial cells (HUVECs). While, EPCs did not form tubuli like structures when cultured alone on the Matrigel™ (data not shown), they exhibited an angiogenic stimulatory effect on the ability of endothelial cells to form tubuli. As shown in Fig. 3B and summarized in Fig. 3D, the early EPCs stimulated the endothelial cells (HUVECs) to form a denser network of tubuli with more branching points and longer tubuli (Fig. 3D). The early EPCs, traced with a cell-tracker green, integrated with the tubuli formation in both the branching points and the tubes (Fig. 3C).

### Adhesion of early EPCs to endothelial cells during static and dynamic flow conditions

**Figure 4:**
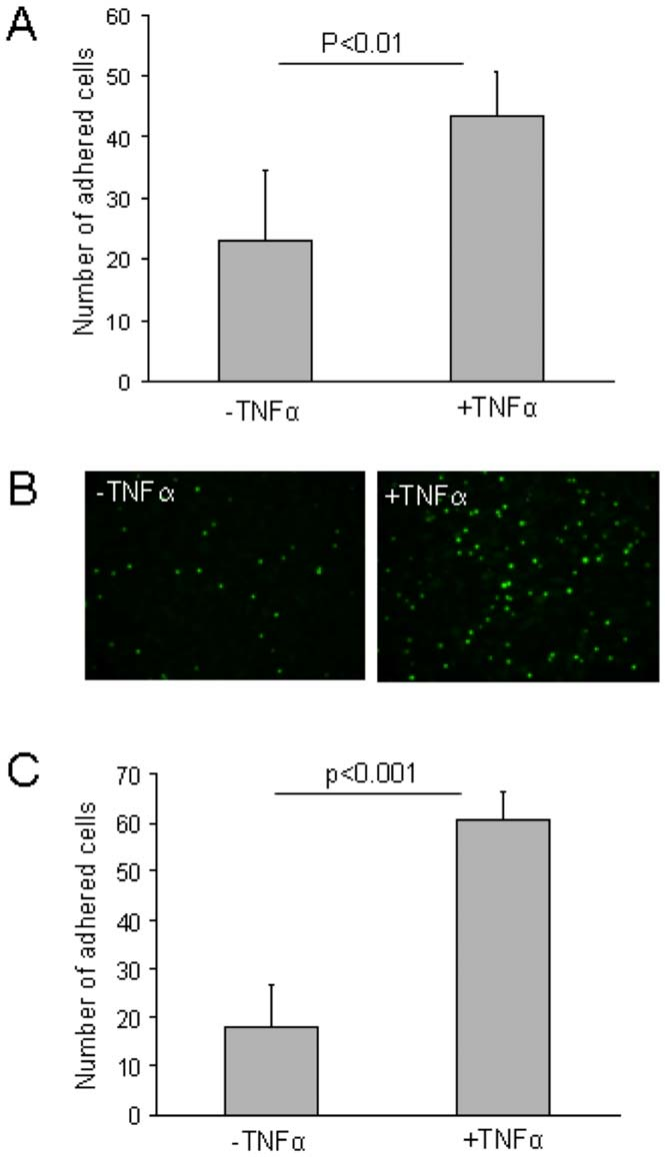
Adhesion of early EPCs to endothelial cells during static conditions and dynamic flow conditions. Adhesion of early EPCs (pre-stained with cell tracker green) to endothelial cells (HUVECs), which are pre-incubated with or without TNFα(10 ng/ml) for one h. **A.** Number of adhering early EPCs to HUVECs after one h of static adhesion assay (mean and SD of n = 3). **B.** Immunofluorescence microscopy of one h static adhesion assay of early EPCs (green) adhered to HUVECs that had been pre-incubated with or without TNFα (10 ng/ml). **C.** A representative flow chamber experiment where the bar graph shows the number of adherent cells after three minutes of flow perfusion (50 s^−1^) of early EPCs into a capillary coated with HUVECs. Mean and SD of n = 3.

Adherence of early EPCs to endothelial cells is a property that may indicate the homing potency of the early EPCs. For the assessment of early EPC adhesion to inflamed endothelium, static adhesion and flow chamber adhesion assays were carried out. Inflamed endothelium was mimicked by pre-treatment of the HUVECs with TNFα (10 ng/ml) for 1 h. In the static adhesion we found that early EPCs adhered to HUVECs and the number of adherent cells was significantly increased when HUVECs were pre-treated with TNFα(22.9±11.5 versus 43.6±7.1, mean and SD, n = 3, p<0.01) (Fig. 4A). Representative photomicrographs of early EPCs, pre-stained with cell-tracker green, adhered to HUVECs in the static adhesion assay are shown in Fig. 4B.

In the dynamic flow chamber adhesion assay performed under continuous flow (50 s^−1^) for three min, the early EPCs did not adhere to fibronectin-coated capillaries (data not shown). However, in capillaries coated with HUVECs, early EPCs adhered to both untreated and TNFα-treated HUVECs (Fig 4C). The number of early EPCs adhering to HUVECs pre-treated with TNFα was significantly higher compared to non-treated HUVECs (18±8.9 versus 60.7±5.5, mean and SD, n = 3, p<0.001).

### Differential gene expression in response to fibronectin mediated differentiation

**Figure 5:**
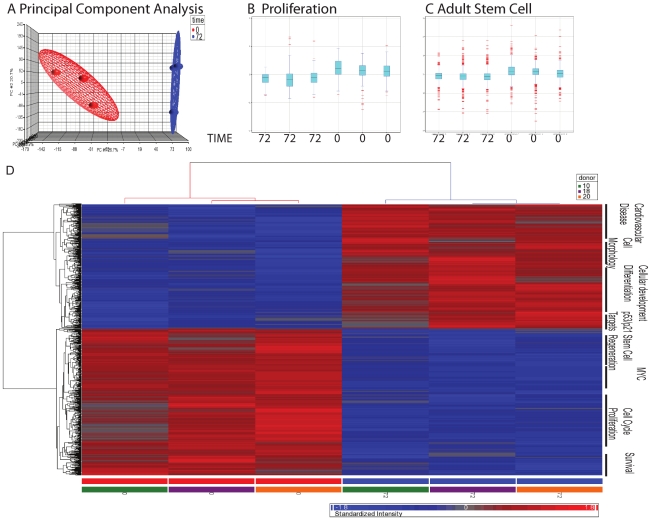
Gene expression profiling before and after 72 hour differentiation on fibronectin. **A.** Principal Component analysis (PCA), an exploratory multivariate statistical technique was used to simplify the complex microarray changes that occur in three individuals (patient 10, 18 and 20) during 72 hours on fibronectin. This is done by reducing the dimensionality of the data matrix by finding r new variables (that sum the expression of multiple genes into single axes), shown here are the average signal of each sample along the three dimensional virtual space of the first three principal components. **B and C.** The box-and-whisker plot describing the distribution of feature intensities. The x-axis represents the individual microarray, while the y-axis represents the feature intensity values. Boxes represent the interquartile range, with the 75th percentile at the top and the 25th percentile at the bottom. The line in the middle of the box represents the 50th percentile, or median, while the plus represents the mean. Whiskers represent the rest of the distribution, with their terminations representing the lowest and highest feature intensity values. Box-and-whisker plots were performed for genes from the gene ontology term “Cell cycle and Proliferation” (**B**) and “adult stem cell” (**C**). **D.** Hierarchical Clustering of 466 differentially expressed genes, plotted according to their degree of respective co-expression. Columns represent samples, while rows represent genes. Gene ontology terms that are tightly co-expressed are listed on the right panel of the cluster. The origins of the sample (time point and patient donor) are listed on the bottom. The degree of correlation between genes (left) or samples (top) are plotted in a tree view fashion.

**Figure 6:**
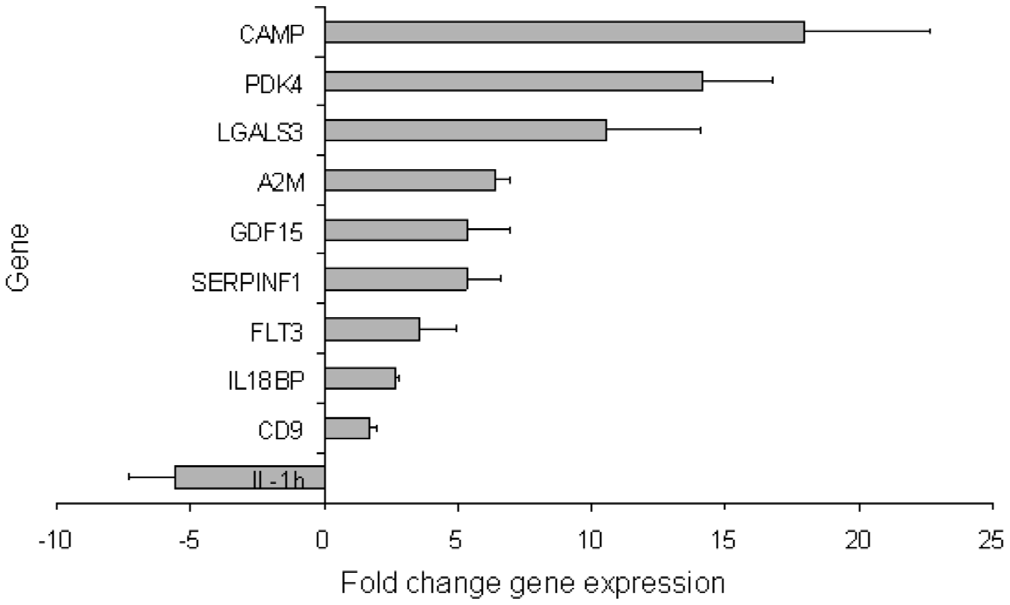
Selection of highly differentially expressed genes and their respective fold change in response to 72 hours of culture on fibronectin. Validation of gene expression by quantitative real time PCR. The mean triplicate gene expression was obtained using differences in cycle threshold between the gene and 18 s (ΔCt). The fold change in difference (ΔΔCt) in gene expression in the compared samples before and after 72 hours culture on fibronectin was determined (2ΔΔCt) and expressed in the diagram as mean and SEM of n = 3.

**Figure 7:**
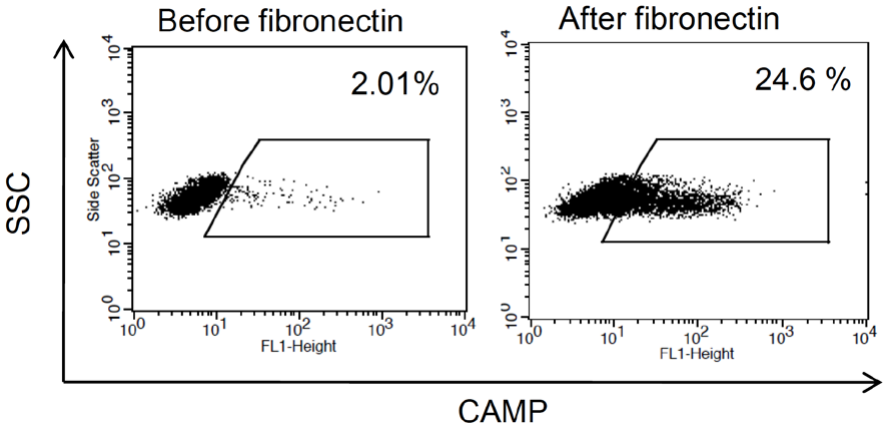
Expression of LL-37 (CAMP) after 72 hours of culture on fibronectin. Depicted is the intracellular protein expression of LL-37 (CAMP) in native cells before (left dot blot) and after (right dot plot) the 72 h differentiation period on fibronectin coated dishes detected by flow cytometry.

The gene expression profiling was done before and after a 72 h culture and differentiation period on fibronectin coated dishes (Fig. 1B last two panels). The microarray was undertaken in three biological repeats (three different cord blood donors). Box-and-Whisker plots confirmed that the normalisation of the arrays achieved comparable dynamic range of the different samples (Figure S1). By contrast, Principal Component Analysis (PCA) demonstrates that the gene expression of these two time points is systematically distinct across the three donors (Fig. 5A). Only those genes that showed at least a fold-fold up- or down-regulation, with a P-value less than 0.05 were considered for further analysis. The significantly up-regulated genes chosen for further analysis based on their functional properties include LL-37 (CAMP), PDK4, LGALS3, A2M, GDF15, SERPINF1, FLT3, IL-18BP, CD9 and IL-1β. These differentially expressed genes and their fold change before and after fibronectin culture are summarized in Tabl. 1. The expression profile of the selected genes in the same three donor samples used for the micro array was confirmed using real-time quantitative PCR (RT-QPCR). The fold change of the gene expression, as determined by RT-QPCR is demonstrated in Fig. 6. In addition the intracellular expression of LL-37 (CAMP), the most up-regulated gene, was assessed by flow cytometry before and after the 72 h culture and differentiation period (Fig. 7). The analysis of early EPCs grown in the presence of fibronectin detected 465 differentially expressed genes, of which 223 were up regulated and 242 down regulated. To benchmark early EPC population differentiation response to existing microarray literature and gain more biological insight from the expression pattern, we have employed Ingenuity Pathways Analysis® and Gene Set enrichment Analysis [24]. Both methods identified enrichment of select Gene Ontology terms, such as adult stem cell and proliferation (Figure 5 B and C), both elevated in the zero time point. A selection of differentially expressed genes with correlation to angiogenesis, differentiation and CVD is described in Table 1. Hierarchical clustering of gene expression further demonstrates that the genes that represent enriched gene sets from previous experiments and Gene Ontologies (data not shown) with specific cellular functions, such as cell differentiation and cell cycle arrest, are frequently tightly co-expressed. The latter further supports the view that 72 h culture on fibronectin induced coordinate programmatic gene expression and differentiation (Fig. 5D).

**Table 1:** A selection of differentially expressed genes from early EPCs cultured on fibronectin for 72 hours.

Gene ID	Gene symbol	Gene name	Fold change	p-value
820	CAMP	cathelicidin antimicrobial peptide	20.40	0.002
5166	PDK4	pyruvate dehydrogenase kinase, isozyme 4	10.30	0.001
2	A2M	alpha-2-macroglobulin	9.30	0.010
7004	GDF15	growth differentiation factor 15	3.76	0.023
10068	IL18BP	interleukin 18 binding protein	3.10	0.002
5176	SERPINF1	serpin peptidase inhibitor, clade F, member 1	2.90	0.008
928	CD9	CD9 molecule	2.90	0.050
3958	LGALS3	lectin, galactoside-binding, soluble, 3	2.64	0.005
2322	FLT3	fms-related tyrosine kinase 3	2.20	0.078
3553	IL-beta	interleukin 1beta	−40.0	0.010

Differentially expressed genes were selected based on a baysian “volcano plot” expression with a fold change greater than 2, and significance P value <0.05, as determined by student's t-test.

## Discussion

The relative scarcity of the circulating cell population that displays the ability to trigger endothelial repair and angiogenesis (traditionally summarized under the general term EPC) is one of the major obstacles limiting the detailed functional analysis and molecular characterization of EPCs [2], [5], [25]. In this paper, we describe a method that allows the generation of a high number of putative early EPCs after *in vitro* differentiation of expanded cord blood derived CD34+ cells. In addition, we provide a functional and gene expression profile of these cells.

Cord blood derived CD34+ cells were firstly expanded for seven days in serum free expansion medium, and then incubated for three days in endothelial cell growth medium. This allowed for the generation of a high number of cells, which could be further differentiated into putative early EPCs when cultured in the presence of fresh endothelial cell growth medium on a fibronectin coated matrix.

We initiated the *in vitro* expansion with CD34+ selected cord blood. This approach was chosen as the vast majority of the flow cytometry based EPC-definition in the literature includes positivity for the cell surface marker CD34 [2], [3], [13]. Furthermore, CD34+ hematopoietic progenitors display pro-angiogenic capacities in animal models and in humans with acute myocardial ischemia [2], [7], [26]. After expansion and induction of the differentiation process, the cells adopted a spindle like shape similar to the first morphological description of EPCs [1] and expressed VEGFR-2 (KDR, CD309) and VE-cadherin (CD144). These surface markers have been used successfully to identify circulating EPCs by flow cytometry [16], [27].

Interestingly, our putative early EPCs strongly expressed the integrin subunits CD61 and CD18. Integrins are heterodimeric transmembrane cell adhesion receptors composed of α- and β-subunits. They play an essential role in processes such as cell migration and homing, as well as cell adhesion and signalling. Our findings of the expression of the β_2_-integrin subunit (CD18) together with the demonstrated adhesion to TNFα-treated HUVECs are in line with previous studies showing a strong expression of β_2_-integrins on EPCs and their role in mediating homing to ischemic myocardium and injured endothelium [28], [29]. The β_3_-integrin subunit (CD61) is either a component of the platelet fibrinogen receptor integrin α_IIb_β_3_ (CD41/CD61) or the vitronectin receptor integrin α_V_β_3_ (CD51/CD61), which is expressed on a variety of different cell types including endothelial cells [30]. Although CD61 expression has been recently linked to hematopoietic progenitor cells [31], integrin α_V_β_3_ has not previously been reported on EPCs. Thus, α_V_β_3_ might be an interesting molecule to be further evaluated as an EPC marker. In addition to the expression of cell surface molecules that have typically been described on EPCs we also found the cells in our model to stain double positive for the uptake of Dil-labelled acetylated LDL and FITC-labelled ulex lectin. This double staining method alone or in conjunction with flow cytometry has been widely used to describe cells as EPCs [3], [5], [13]. The high cell number generated by our in vitro expansion and differentiation method allowed us to perform additional functional characterization of our early EPCs via endothelial tube formation and colony forming unit assays. The results of both assays support the notion that our *in vitro* generated early EPCs are functionally active. In addition recent reports by other groups at least partially confirm a similar phenotype and functional activity of either CD133+ or CD34+ selected cord blood derived progenitor cells after in vitro expansion of this rare cell type [32], [33].

The usage of fibronectin-coated plates for culturing of peripheral blood derived mononuclear cells was one of the key steps that led to the initial discovery of EPCs [1]. Since then fibronectin has been used to generate EPCs from non-selected mononuclear cells or CD34+ and CD133+ hematopoietic progenitors. However, to the best of our knowledge a detailed examination addressing the impact of fibronectin-induced cell differentiation by gene expression analysis has not yet been reported. Most of the highly up-regulated genes that we identified have not been described in relation to the differentiation of early EPCs before. A recent study reports differential gene expression between early EPCs and outgrowth endothelial cells (OECs) [34] also known as endothelial colony forming cells (ECFC) [3] with the same gene expression bead chips, comprising more than 48,000 human transcripts, that were used in our study. None of the genes identified in our approach was described in the comparison of early EPCs versus OECs. This could be due to the fact that mononuclear cells (MNCs) were used instead of CD34+ selected cells as we did in our study. However, it is more likely explained by the two different culture methods that had been used for the generation of early EPCs versus OECs. Fibronectin was used for the differentiation of MNCs to early EPCs and collagen-coating was used for the differentiation of MNCs to OECs [34]. Whereas our approach was based on the analysis of the same cell population (originating from CD34+ selected cord blood MNCs) at different time points, before and after a 72 h fibronectin-induced differentiation period.

Expression profiling is a powerful tool both in characterization of cells as well as in its use as a discovery tool. Many of the genes found to be up-regulated are major players in angiogenesis and/or are expected to mediate atheroprotective effects, which are properties that have been attributed to early EPCs. The list of newly identified genes, up-regulated both in mRNA expression profiling as well as in quantitative PCR, include cathelicidin antimicrobial peptide (CAMP, LL-37), pyruvate dehydrogenase kinase isozyme 4 (PDK4), alpha-2-macroglobulin (A2M), growth differentiation factor 15 (GDF15), serpin peptidase inhibitor clade F (SERPINF1, PEDF) and galectin-3 (LGALS3).

The gene identified to be most up-regulated was LL-37 (CAMP). This secreted 18 kDa peptide belongs to the group of antimicrobial peptides that inherit functions in the innate immune system, inflammation and angiogenesis. It is up-regulated in differentiating cells [35] and it induces cell proliferation via the P2X_7_ receptor [36]. Furthermore, LL-37 (CAMP) has a pro-angiogenic effect by directly acting on endothelial cells and inducing neovascularization [37]. Our observation of highly up-regulated expression of LL-37 (CAMP) after the differentiation of EPCs on fibronectin coated dishes is in line with the reported pro-angiogenic functions [37]. The fact that LL-37 (CAMP) is up-regulated in early EPCs generates the hypothesis that it contributes to the paracrine pro-angiogenic effect of early EPCs. Interestingly, a recent study demonstrated LL-37 (CAMP)-induced recruitment of embryonic (e) EPCs to ischemic tissue by up-regulation of the adhesion molecule PSGL-1 (CD162) on eEPCs [38], this is in line with our findings (data not shown) of increased expression of PSGL-1 on *in vitro* differentiated early EPCs. Furthermore, a very recent report indicates that LL-37 (CAMP) is directly involved in differentiation processes (in this case the *in vitro* differentiation of monocytes towards a more immature osteoblast/osteoclast-like cell type) [39].

Another pro-angiogenic gene, that we found to be up-regulated, was the carbohydrate-binding protein galectin-3 (LGALS3), which has a molecular weight of 35 kDa. Galectin-3 is involved in cell-cell interactions and cell signalling and acts pro-angiogenic, presumably related to its ability to bind laminin and fibronectin as well as to stimulate the expression of α_V_β_3_ integrins (CD51/CD61) and to induce migration of endothelial cells [40], [41]. Furthermore, it exerts anti-atherosclerotic effects via removal of oxidized LDLs [42]. Our finding that galectin-3 is up-regulated on EPCs is in line with a previous report demonstrating that galectin-3 is expressed on a higher level in EPCs at day 14 of *in vitro* differentiation as compared to its expression level in HUVECs, LMECs or AoECs [43]. The extent of up-regulation of CAMP and LGALS3 indicates that these genes are major players in EPC differentiation.

SERPINF1, also known as pigment epithelium-derived factor (PEDF), is a 50 kDa secreted glycoprotein that belongs to the family of serin protease inhibitors (Serpins). An atheroprotective function of SERPINF1 has been reported [44]. It reduces angiotensin-II-induced endothelial cell activation through Nox-4, blocks TNFα-induced stimulation of endothelial cells and inhibits neointimal hyperplasia by limiting proliferation and migration of smooth muscle cells after balloon injury and it accelerates re-endothelialization [43], [44]. These effects may contribute to the vessel protective functions of EPCs and up-regulation of SERPINF1 may constitute a major part of EPC differentiation.

We also identified GDF15 and alpha-2-macroglobulin among the up-regulated genes in response to the fibronectin-induced differentiation of EPCs. GDF15 is a secreted disulfide-linked dimeric protein with a molecular weight of 25 kDa that belongs to the transforming growth factor-β superfamily [45]. It has been shown to elicit cardioprotective effects in ischemia/reperfusion injury [46] presumably via anti-inflammatory and anti-apoptotic effects [45]. The association of GDF15 with early EPC function is novel and warrants further investigations in particular based on the accumulating evidence of GDF15 as a cardiovascular biomarker predicting cardiovascular adverse events in patients with acute coronary syndromes [47], [48]. Furthermore, very recently anti-inflammatory properties have been attributed to GDF15 [49]. Alpha-2-macroglobulin (A2M) is a tetramer of four identical subunits, each of them 185 kDa. A2M is found at high levels in human plasma and binds to a variety of cytokines including TGF-β and PDGF, these complexes are cleared from the circulation by binding of A2M to the LDL receptor-related protein [50]. A recent proteomics study identified A2M in the secretome of EPCs [51]. Together with our expression profiling and quantitative PCR data this implies a potential role of A2M in EPC differentiation.

In conclusion, we describe a method for the generation of large numbers of cord blood-derived early EPCs and demonstrate the functional capacity and phenotypic characterization of these stem cells. In addition, we use gene expression profiling to provide further support for cell differentiation towards early EPCs and at the same time use the discovery potential of this technology to identify genes involved in EPC differentiation. Our data confirm that CD34+ hematopoietic progenitors are capable to differentiate into early EPCs. Generating sufficient numbers of well characterized cord blood-derived early EPCs will facilitate pharmacological screening and detailed characterization of drugs/factors that influence early EPC function. Particularly the development of anti-atherosclerotic drugs may benefit from such screening approaches. Furthermore, the newly identified, highly up-regulated genes, in particular LL-37 (CAMP), may direct research towards a better understanding of the molecular program of EPC differentiation and towards the development of new EPC-based therapeutic strategies.

## Supporting Information

Figure S1 **Distribution of gene expression signal intensities >250.** The box-and-whisker plot to examine the distribution of feature intensities for 6,064 genes that exhibited higher than 250 raw intensity in at least three samples across all microarrays. 1,2 and 3 represent cord-blood donors. A and B represent after and before 72 hours culture on Fibronectin.
(TIF)

Figure S2 **The viability of the cells during the culture procedure.** The percentage of apoptotic cells as assessed by FITC-labelled Annexin V during the 13 days of expansion and differentiation of EPCs, i.e after seven days expansion, after further three days expansion, and finally after three days differentiation on fibronectin. The histograms show the percentage of apoptotic Annexin V cells of one representative donor that was followed during the expansion and differentiation procedure.
(TIF)
