# Supplementary figures and images for: Successful In Vitro Expansion and Differentiation of Cord Blood Derived CD34+ Cells into Early Endothelial Progenitor Cells Reveals Highly Differential Gene Expression
Source: PLoS One. 2011 Aug 12;6(8):e23210. doi: 10.1371/journal.pone.0023210 (PMC3155543; doi:10.1371/journal.pone.0023210)

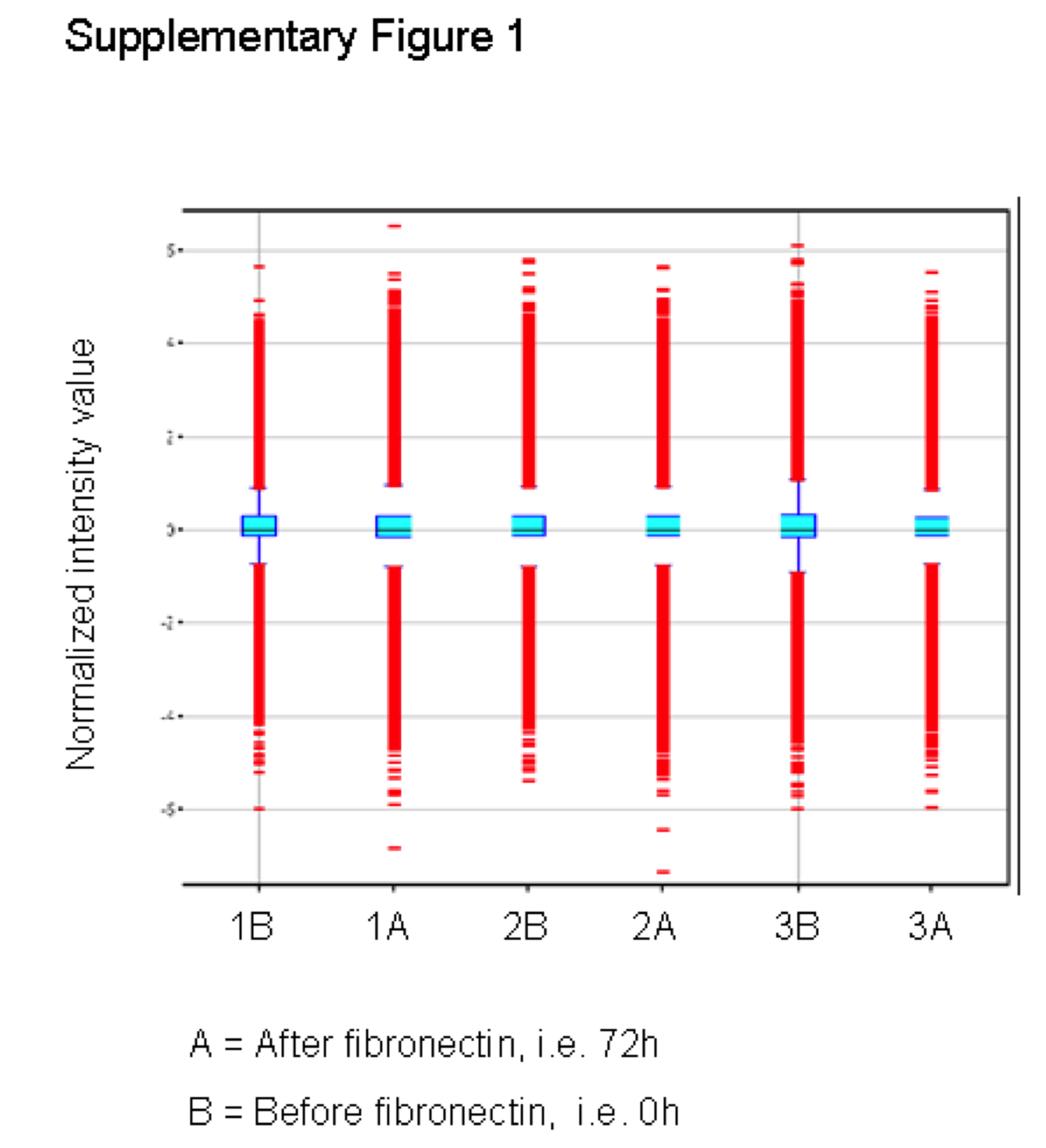

Supplement: Figure S1 — Distribution of gene expression signal intensities >250. The box-and-whisker plot to examine the distribution of feature intensities for 6,064 genes that exhibited higher than 250 raw intensity in at least three samples across all microarrays. 1,2 and 3 represent cord-blood donors. A and B represent after and before 72 hours culture on Fibronectin. (TIF) [file pone.0023210.s001.tif]

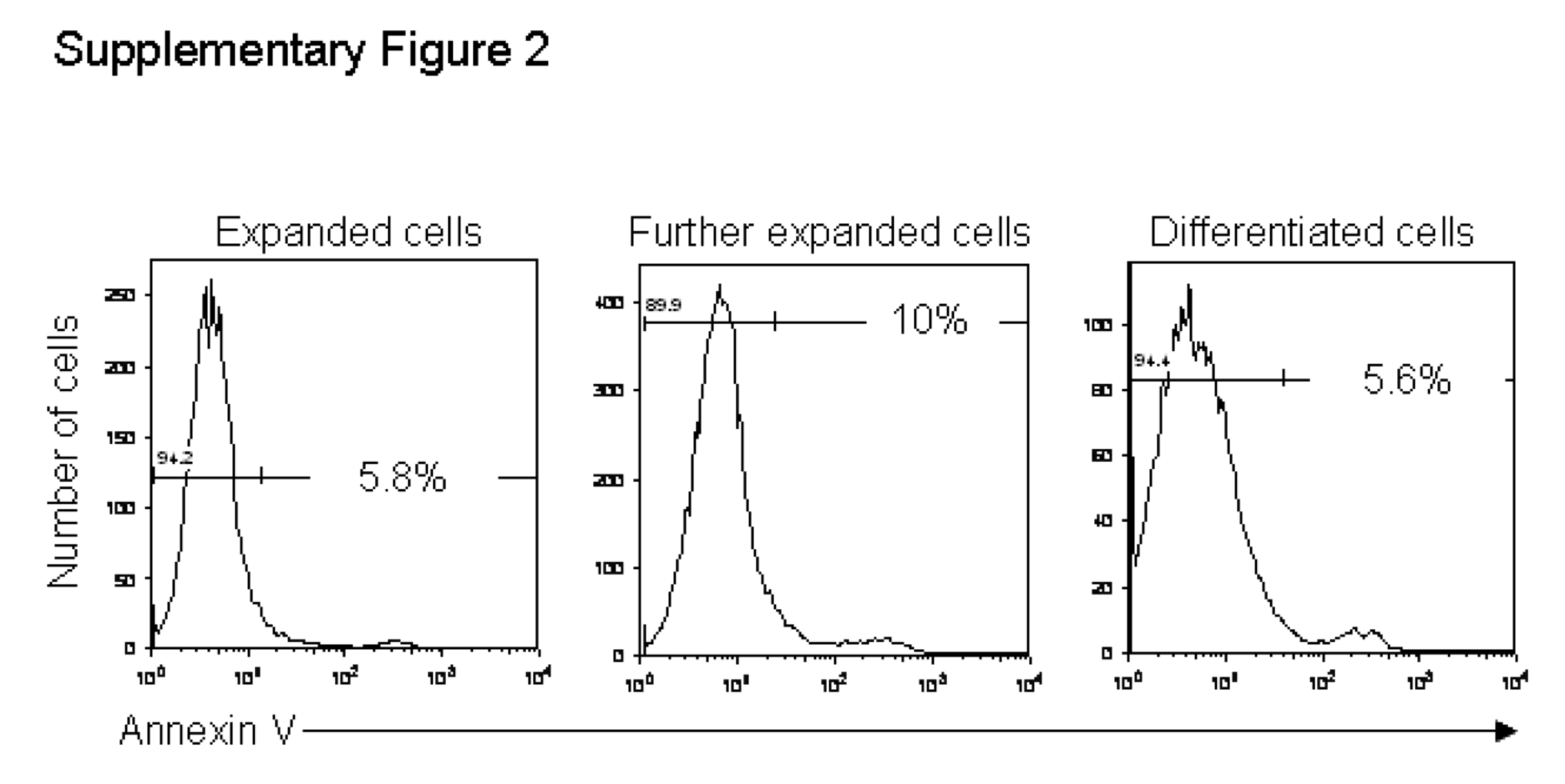

Supplement: Figure S2 — The viability of the cells during the culture procedure. The percentage of apoptotic cells as assessed by FITC-labelled Annexin V during the 13 days of expansion and differentiation of EPCs, i.e after seven days expansion, after further three days expansion, and finally after three days differentiation on fibronectin. The histograms show the percentage of apoptotic Annexin V cells of one representative donor that was followed during the expansion and differentiation procedure. (TIF) [file pone.0023210.s002.tif]
